# Temperature-Dependent Demographic Characteristics and Control Potential of *Aphelinus asychis* Reared from *Sitobion avenae* as a Biological Control Agent for *Myzus persicae* on Chili Peppers

**DOI:** 10.3390/insects11080475

**Published:** 2020-07-27

**Authors:** Sheng Yin Wang, Bo Li Wang, Gai Lan Yan, Yan Hong Liu, Da Yu Zhang, Tong Xian Liu

**Affiliations:** 1 College of Agriculture and Food Science, Zhejiang A&F University, Hangzhou 311300, China; 20160040@zafu.edu.cn (S.Y.W.); zhangdayu@zafu.edu.cn (D.Y.Z.); 2 College of Plant Protection, Northwest A&F University, Yangling 712100, China; liuyanhong1984@126.com; 3 College of Economic and Management, Zhejiang A&F University, Hangzhou 311300, China; zafuwlb@163.com; 4 Fengdong New City Administration for Market Regulation, Xi’an 710086, China; yglnafu@163.com; 5 College of Plant Protection, Shanxi Agricultural University, Taigu 030801, China

**Keywords:** biological control, green peach aphid, chili pepper, leaf disc, life table

## Abstract

*Aphelinus asychis*, a polyphagous parasitoid, has been widely used as an efficient biological control agent against the aphid *Myzus persicae.* Aiming to evaluate the influence of temperature on the biological characteristics and control potential of *A. asychis* for *M. persicae*, we compared the life table parameters and control potential of *A. asychis*, which included the developmental time, longevity, fecundity, intrinsic rate of increase (*r*), and finite killing rate (*θ*). The results showed that increasing the temperature significantly decreased the developmental time and longevity of *A. asychis*. The *r* at 24 (0.2360 d^−1^) and 28 °C (0.2441 d^−1^) were significantly greater than those at 20 (0.1848 d^−1^) and 32 °C (0.1676 d^−1^). The *θ* at 24 (0.4495), 28 (0.5414), and 32 °C (0.4312) were also significantly greater than that at 20 °C (0.3140). The relationship between population fitness (*r* and *θ*) and temperature followed a unary quadratic function (*R*^2^ > 0.95). The temperatures for the expected maximum intrinsic rate of increase (*r*_max_) and the maximum finite killing rate (*θ*_max_) were 25.7 and 27.4 °C, respectively. In conclusion, *A. asychis* could develop and produce progenies within the temperature range of 20–32 °C, and its control efficiency for *M. persicae* at 24, 28, and 32 °C was greater than that at 20 °C. The most suitable temperature range for controlling *M. persicae* with *A. asychis* in the field might be between 25.7 and 27.4 °C.

## 1. Introduction

The chili pepper (*Capsicum annuum*, Solanaceae) is an important vegetable and condiment planted in greenhouses and open-air fields in China [1]. The green peach aphid (*Myzus persicae*) is a sucking pest of more than 400 host plant species covering 40 families, including the chili pepper [2,3]. It is also an important vector of more than 100 plant viruses [2]. Its fast development and high fecundity promote the build–up of large populations within a short period, especially in greenhouse agroecosystems [4]. In the past few decades, the intensive use of chemical insecticides such as carbamate [5], pyrethroid [6], cyclodiene [7], neonicotinoid [8], and many others to control *M. persicae* has resulted in their development of resistance to these chemicals. Furthermore, chemical insecticides also have tremendous negative impacts on beneficial organisms and the environment [9,10,11].

Because of the critical economic damage by *M. persicae* to chili peppers, many biological agents, including predators and parasitoids, have been extensively studied and used, especially aphelinidae wasps [12,13,14]. There are 84 species in the genus *Aphelinus* worldwide [15,16]. Most species in this genus play important roles in the biological control of aphids and have been widely used in vegetable and fruit production in greenhouses and in the field [17,18]. Among them, *Aphelinus asychis* is a polyphagous endoparasitoid of about 40 aphid host species including *M. persicae* and *Sitobion avenae* [13,18,19,20], and it has been used under field and greenhouse conditions [21,22].

In biological control, banker plant systems have been extensively used to control vegetable pests, including *M. persicae* [12,23,24,25]. A typical banker plant system includes three important elements: a banker plant, alternative host, and natural enemy [12]. The cost of the winter wheat (*Triticum aestivum*) plant is low, and the cultivation and management techniques are relatively simple. In addition, the host plants of *S. avenae* are only gramineous crop and grass, including the winter wheat plant [26]. Therefore, the wheat plant and *S. avenae* were suitable to combine for the banker plant system in many previous studies [13,27]. In this research, we used the winter wheat plant *S. avenae* and *A. asychis* to form a banker plant system, which was a continuation of our previous study [13,14].

In this banker plant system, the alternative host and natural enemy are insects, and both are ectotherms. Their physiological functions, such as locomotion, feeding, and population fitness, are significantly affected by many environmental factors, especially temperature [28,29,30]. In addition, the geographical distribution and invasion range of the insects is also limited by environmental temperature [31,32]. Therefore, the influence of temperature on the control efficiency and population fitness of many natural enemies has been estimated in many previous studies, such as of *Aphidius gifuensis*, *Encarsia Formosa*, *Ophraella communa*, and *Harmonia dimidiata* [33,34,35,36]. 

The control efficiency of parasitoids is influenced by many factors, such as temperature, prey species, host plants, etc. [37]. The linkage of the life table, parasitism, and feeding rate showed that the host plant affected the demography and parasitic effectiveness of *A. asychis* against *M. persicae* [13]. Additionally, the population fitness of the parasitoids was affected by temperature [38,39]. However, studies on the influence of temperature on the population fitness and control efficiency of *A. asychis* parasitizing *M. persicae* on chili peppers are lacking. In order to estimate its population fitness and quantitatively assess its control efficacy, we collected and analyzed data on the life table parameters, parasitism rate, and feeding rate of *A. asychis* at four constant temperatures using the age-stage, two-sex life table. In addition, we used population projection to quantitatively predict its killing potential, which might be beneficial to increase the control efficiency of *A. asychis* by regulating the environmental temperature.

## 2. Materials and Methods

### 2.1. Plant and Insect Cultures

The chili pepper plants (var. “Ox horn”) were grown in a soil mixture (peat moss: perlite = 3:1) in plastic pots (10 cm in diameter) and enclosed in nylon net cages (60 × 60 × 60 cm^3^). *Myzus persicae* were reared on chili pepper plants (90 days old), and *A. asychis* was fed on *S. avenae*, which fed on winter wheat plants (variety, “Xinong 979”). The winter wheat plant, the chili pepper plant, *M. persicae*, *S. avenae*, and *A. asychis* were reared in a phytotron (photoperiod, 14:10 h (L/D); illumination intensity, 10,000 ± 50 lx; 25 ± 0.5 °C; and 70 ± 10% RH (Relative Humidity)) at Northwest A&F University, Shaanxi, China. 

### 2.2. Life Table, Parasitism, and Host Feeding

Data on fecundity, the host feeding of *A. asychis*, and its killing of aphids were obtained at 20, 24, 28, and 32 °C following the method described by Wang et al. [13]. About 100 s-instar *M.* *persicae* nymphs were reared on a circular chili pepper leaf disc in a Petri dish (3 cm in diameter), and water-agar (1%) was used to keep the leaf fresh. Ten mated *A. asychis* female adults that had previously emerged from *S. avenae* were transferred into the Petri dish and removed 24 h later. The parasitized and healthy aphid nymphs were maintained in the phytotron at 20, 24, 28, and 32 °C, respectively. After seven days, 50 mummified aphids were randomly selected for each treatment and monitored daily. Emerged *A. asychis* female and male adults were paired. If the male parasitoid was not enough or died before the female, other male adults from the non-tested population were used. Each parasitoid pair was transferred into a new Petri dish containing the fresh chili pepper leaf and 50 s-instar *M. persicae* nymphs for parasitism and feeding. The developmental time, longevity, parasitism, and host feeding of *A. asychis* were recorded until all the tested parasitoids died. 

According to the age-stage, two-sex life table theory, the population parameters of *A. asychis* were analyzed with the computer program TWOSEX–MSChart [40,41,42]. The data on host feeding, non-effective parasitism, and aphid killing were analyzed using the computer program CONSUME–MSChart [43,44]. Using data on the age-stage-specific fecundity (*f_xj_*) and age-stage-specific aphid killing rate (*p_xj_*) at age *x* and stage *j*, the population growth and killing potential of *A. asychis* parasitizing second-instar *M. persicae* nymphs were analyzed using the computer program TIMING-MSChart [45]. The parameter definitions and formulas used in this study are presented in Table 1.

The standard errors of the population parameters, host feeding, non–effective parasitism, and aphid killing were estimated with the bootstrap technique, and the differences among the four constant temperature treatments were also analyzed using the same technique [46,47].

## 3. Results

### 3.1. Life Table

**Figure 1:**
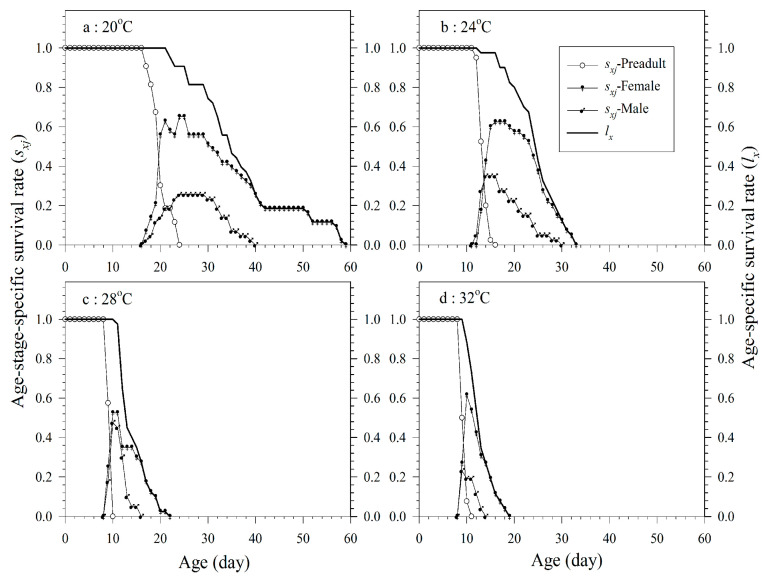
Age-stage-specific survival rates (*s_xj_*) and age-specific survival rates (*l_x_*) of *A. asychis* parasitizing *M. persicae* at 20 °C (**a**), 24 °C (**b**), 28 °C (**c**) and 32 °C (**d**).

Overlaps between stages revealed the different developmental rates among the *A. asychis* individuals. The age-specific survival rate (*l_x_*) of *A. asychis* decreased gradually with increasing age. In the parent cohort, 43, 40, 40, and 26 parasitoids successfully emerged as adults at 20, 24, 28, and 32 °C, respectively. The emergence rates of the parent cohort were 86%, 80%, 80%, and 52% at 20, 24, 28, and 32 °C, respectively. The female proportion in the 20 °C treatment was significantly greater than that in the 28 °C treatment. The increase in temperature caused a significant decrease in the developmental time of *A. asychis.* Its adult longevity, likewise, showed the same trend (Figure 1 and Table 2).

**Figure 2:**
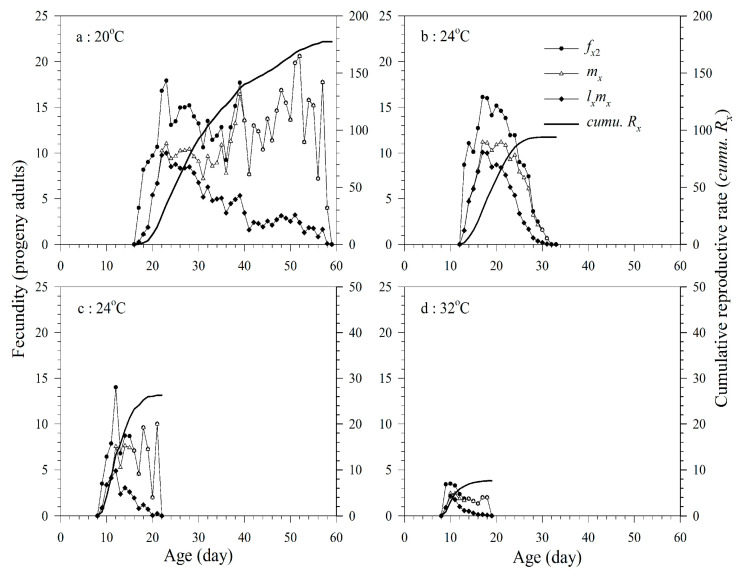
Age-stage-specific fecundity (*f_x_*_2_), age-specific fecundity (*m_x_*), age-specific net fecundity (*l_x_m_x_*), and cumulative reproductive rates (*cumu. R_x_*) of *A. asychis* parasitizing *M. persicae* at 20 °C (**a**), 24 °C (**b**), 28 °C (**c**) and 32 °C (**d**).

The *f_xj_* curve indicates the number of progeny adults produced by the female at age *x* and stage *j*, and *f_x_*_2_ indicates that the female adult is of the second life stage. The *f_x_*_2_, *m_x_*, and *l_x_m_x_* of the female adult showed irregular fluctuations in all treatments. Increasing temperatures significantly decreased the reproduction period. The total number of progeny adults showed a similar trend (Figure 2 and Table 2).

### 3.2. Population Parameters

The values of *r* and *λ* of *A. asychis* at 24 and 28 °C were significantly greater than those at 20 and 32 °C, respectively, while the *R*_0_ and *T* decreased significantly with increasing temperature (Table 3). 

### 3.3. Host Feeding

**Figure 3:**
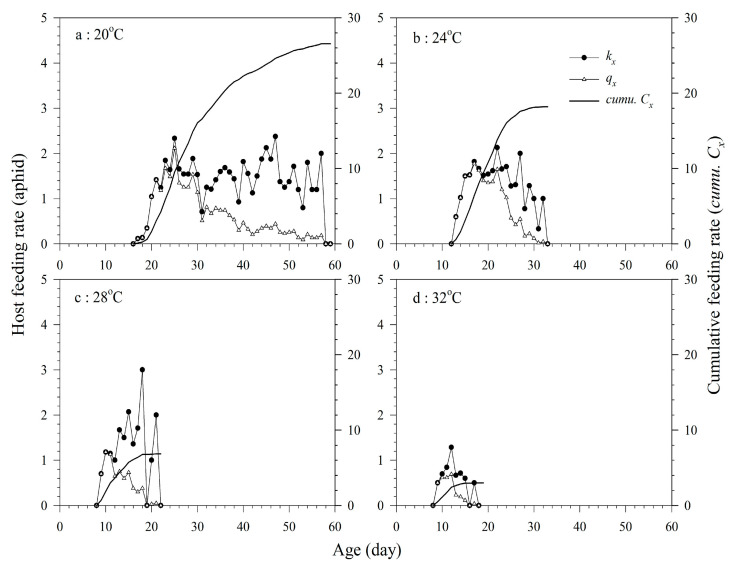
Age-specific host feeding rates (*k_x_*), age-specific net host feeding rates (*q_x_*), and cumulative host feeding rates (*cumu. C_x_*) of *A. asychis* parasitizing *M. persicae* at 20 °C (**a**), 24 °C (**b**), 28 °C (**c**) and 32 °C (**d**).

The *Aphelinus asychis* eggs, larvae, and pupae are in host bodies all the time, and this host is regarded as the parental parasitism. Therefore, the host feeding rate dose not exist before the female adult stage. All the *k_x_* of the adult female of *A. asychis* showed irregular undulation at 20, 24, 28, and 32 °C. The maximum daily *k_x_* at 20, 24, 28, and 32 °C were 2.4, 2.1, 3.0, and 1.3 aphids at ages 47, 22, 18, and 12 d, respectively. The maximum daily values of *q_x_* at 20, 24, 28, and 32 °C were 2.1, 1.8, 1.2, and 0.7 aphids at ages 25, 17, 10, and 12 d, respectively. Increasing the temperature decreased the *C*_0_ of the aphids that were killed by *A. asychis* (Table 3 and Figure 3).

### 3.4. Non-Effective Parasitism

**Figure 4:**
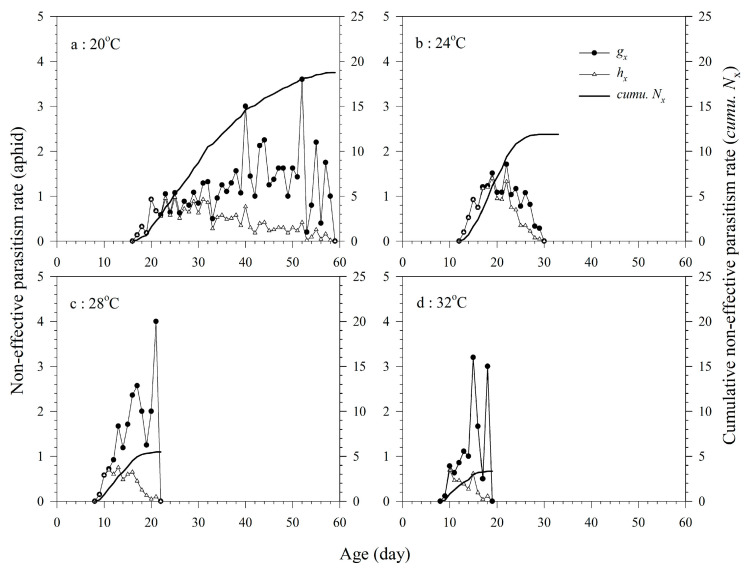
Age-specific non-effective parasitism rates (*g_x_*), age-specific net non-effective parasitism rates (*h_x_*), and cumulative non-effective parasitism rates (*cumu*. *N_x_*) of *A. asychis* parasitizing *M. persicae* at 20 °C (**a**), 24 °C (**b**), 28 °C (**c**) and 32 °C (**d**).

Because *A. asychis* could not parasitize aphids during the pre-adult stage, there was no non-effective parasitism rate before adult emergence. The *g_x_* of *A. asychis* showed irregular fluctuation in all treatments. The maximum daily *g_x_* at 20, 24, 28, and 32 °C were 3.6, 1.7, 4.0, and 3.2 aphids at ages 52, 22, 21, and 15 d, respectively. The maximum daily values of *h_x_* at 20, 24, 28, and 32 °C were 1.0, 1.4, 0.8, and 0.7 aphids at ages 25, 19, 13, and 10 d, respectively. Increasing the temperature significantly decreased the *N*_0_ of *A. asychis* (Table 3 and Figure 4). 

### 3.5. Aphid Killing Rate

**Figure 5:**
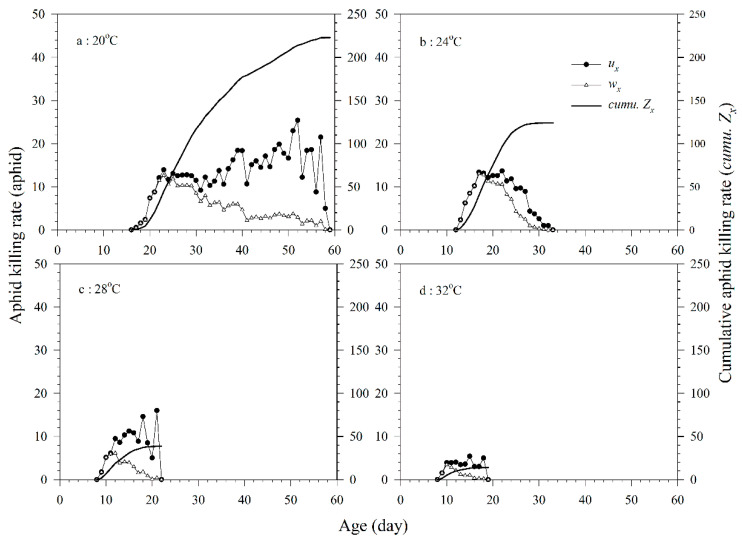
Age-specific aphid killing rates (*u_x_*), age-specific net aphid killing rates (*w_x_*), and cumulative killing rates (*cumu. Z_x_*) of *A. asychis* parasitizing *M. persicae* at 20 °C (**a**), 24 °C (**b**), 28 °C (**c**) and 32 °C (**d**).

Because immature *A. asychis* could not parasitize and feed on aphids, its killing rate during the pre-adult stage could not be determined. The daily *u_x_* of the adult females of *A. asychis* showed irregular undulation at 20, 24, 28, and 32 °C. The maximum *u_x_* at 20, 24, 28, and 32 °C were 25.4, 13.7, 16.0, and 5.4 aphids, respectively. The maximum daily values of *w_x_* in the 20, 24, 28, and 32 °C treatments were 12.6, 13.0, 6.2, and 3.5 aphids, respectively. The *Z*_0_ of aphids by *A. asychis* were 222.8, 124.0, 38.6, and 14.0 aphids per individual at 20, 24, 28, and 32 °C, respectively. The *θ* at 20, 24, 28, and 32 °C were 0.3140, 0.4495, 0.5414, and 0.4312, respectively. The *Q_p_* value of *A. asychis* increased significantly with increasing temperature (Figure 5 and Table 3).

### 3.6. Relationship between Population Fitness and Temperatures

**Figure 6:**
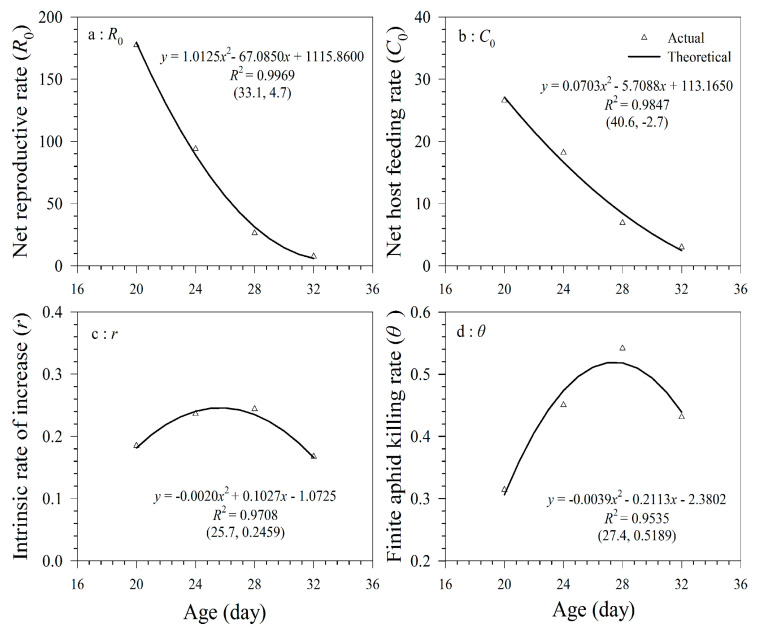
Relationship between population fitness and temperature of *A. asychis* parasitizing *M. persicae* at 20 °C (**a**), 24 °C (**b**), 28 °C (**c**) and 32 °C (**d**).

The relationship between population fitness (*R*_0_, *C*_0_, *r*, and *θ*) and temperature is shown in Figure 6. The relationship between population fitness (the net reproductive rate, net feeding rate, intrinsic rate of increase, and finite aphid killing rate) and temperature followed a unary quadratic function as evidenced by the high coefficient of determination (*R*^2^), greater than 0.95. The net reproductive rate and net host feeding rate decreased as the temperature increased within the range 20 to 32 °C. The temperature for the expected maximum intrinsic rate of increase (25.7 °C) was lower than that for the maximum finite killing rate (27.4 °C).

**Figure 7:**
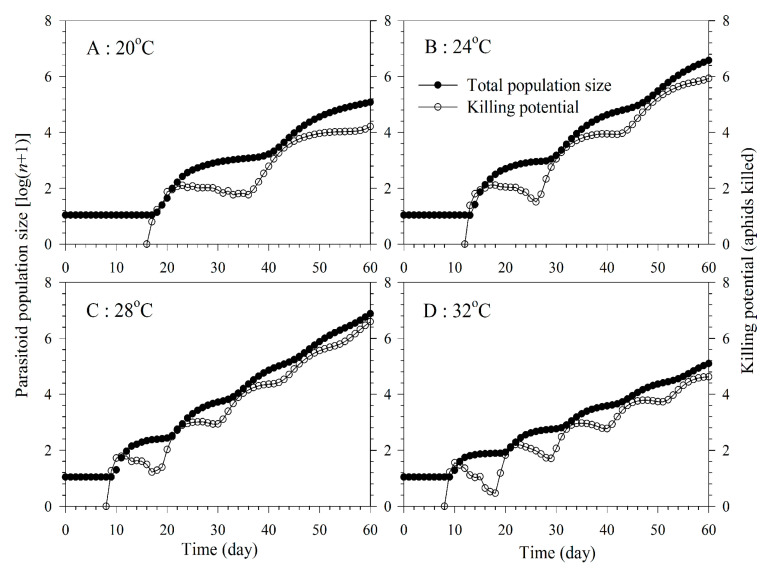
Simulated population growth and killing potential of *A. asychis* parasitizing *M. persicae* at 20 °C (**a**), 24 °C (**b**), 28 °C (**c**) and 32 °C (**d**).

The population projection showed that *A. asychis* increased much faster at 24 and 28 °C (Figure 7). Because the *A. asychis* female does not feed on another host before the adult stage, and male adult does not feed on aphid, the trend of the total population size was different from that of the killing potential. The curve of the female population size showed, however, a similar trend to that of the killing potential. 

## 4. Discussion

Temperature is a vital factor that affects the population fitness of insects, of which the optimal for various insect species may vary [48,49]. In this study, the population fitness of *A. asychis* was evaluated at four constant temperatures. *Aphelinus asychis* could survive and produce progenies at all four temperatures, but higher fitness (*r* and *θ*) was observed at moderate temperature (24 and 28 °C). The fitting of the data to a unary quadratic function showed that the temperatures for the expected maximum intrinsic rate of increase (*r*_max_) and the maximum finite killing rate (*θ*_max_) were 25.7 and 27.4 °C, respectively. In addition, the temperature for the *r*_max_ of *M. persicae* was between 20 and 25 °C [50]. Thus, we inferred that the best temperature range for controlling *M. persicae* with *A. asychis* as a biological agent in chili pepper fields might be 25.7–27.4 °C. 

Numerous factors might affect the developmental time of *A. asychis.* Aphelinid wasps, in general, have a developmental time of 15–30 days [13,51,52,53]. Specifically, the developmental times of *A. asychis* at 23.9 and 32.2 °C have been determined to be 16 and 10 days, respectively [54]. We found that increasing the temperature significantly decreased the developmental time of the *A. asychis* female and male. The developmental duration of *A. asychis* females and males was significantly affected by host age when it fed on *Aphis gossypii*, which was 14.5 d and 14.4 d in 1–2 day old *A. gossypii*-nymphs, 13.5 d and 13.1 d in 4–5 day old nymphs, and 12.3 d and 12.2 d with *A. gossypii* adults as the hosts at 25 °C, respectively [53]. Additionally, the developmental times of the *A. asychis* female and male from the egg to the adult stage and parasitization of *A. gossypii* at 25 °C were 13.9 and 13.2 d, respectively [55]. When it parasitized *A. gossypii* at 20, 25, and 30 °C, the developmental times were 20.6, 14.2, and 13.0 d respectively [56]. Differences among these parameters may be attributed to the temperature, host species, and host stage.

In previous studies, both host species and stage were reported to affect the proportions of *A. asychis* female adults [54,57]. When the *Schizaphis graminum* nymph was used as a host, the older-aged nymphs produced a higher proportion of *A. asychis* female progenies [58]. The proportions of *A. asychis* that parasitized 1–2-day-old and 4–5-day-old *A. gossypii* nymphs and adults were 47.4%, 41.2%, and 47.7%, respectively [53]. Additionally, the proportion of female adults produced by *A. asychis* parasitizing a combination of second and third instar *A. gossypii* nymphs was 51.9% [55]. The temperature under which the parasitoids are reared may also affect sex ratio. For instance, the highest portion of females on *Diaeretiella rapae* was 70% at 7.2 °C, and the lowest was 50% at 29.4 °C [59]. Kang et al. reported that the percentages of female adults were 71.7%, 65.0%, and 78.8% at 20, 25, and 30 °C, respectively [56]. This was similar to our results. 

Many biotic and abiotic factors, including the host species, host plant, and temperature, could affect the longevity of *A. asychis.* For example, the longevity of *A. asychis* female adults when they parasitized *S. graminum*, *Rhopalosiphum maidis*, or *Sipha flava* was similar with some variation—about 18 days under greenhouse conditions [54], 20 days when they parasitized *S. graminum* under field conditions [57], 21 days when they parasitized the second and third instar nymphs of *A. gossypii* at 25 °C [55], and 23 days with second instar nymphs of *M. persicae* on chili peppers at 25 °C [13]. In this study, we found that increasing the temperature significantly decreased the total longevity of *A. asychis*, and the adult longevities of the females and males showed similar responses to temperature.

In this study, the number of progeny adults of the parasitoids decreased significantly as the temperature increased. When they parasitized *S. graminum*, *R. maidis*, and *S. flava* at 23.9, 26.7, 29.4, and 32.2 °C, the number of *A. asychis* progeny was less than 200 [54]. *A. asychis* females produced 232.3, 44.7, and 21.1 eggs when they parasitized 1–2-day-old *A. gossypii* nymphs, adults, and 4–5-day-old nymphs, respectively [53]. When *A. gossypii* was used as a host, *A. asychis* females produced an average of 342.9 mummified aphids at 25 °C [55], which was more than that at 24 °C in this study. When it parasitized the second instar nymph of *M. persicae* on chili peppers at 25 °C, each *A. asychis* female produced more eggs (414.6) than that (238.6 eggs) recorded at all four constant temperatures in this study [13]. The difference between these studies may be due to many factors, including the host species, parasitoid strains, or host plants. 

Moreover, the *R*_0_ of *A. asychis* in this study showed a significant decrease with a temperature increase, which indicated that the *R*_0_ was negatively influenced by temperature. The regression equation for *R*_0_ and temperature supports this phenomenon. In this study, the temperature for the expected maximum intrinsic rate of increase (25.7 °C) was lower than that for the maximum finite killing rate (27.4 °C). This shows that the different population characteristics (i.e., population growth and parasitism rate) may respond differently to environmental factors.

The host feeding behaviors of *A. asychis* on some host species have been previously studied. It was noted that aphids fed on by *A. asychis* females were first paralyzed and usually died after feeding [18]. In addition, *A. gossypii* nymphs and adults were acceptable for host feeding by *A. asychis*, and the number of younger instar aphids for host feeding was higher than that of older instars [53]. They speculated that older aphids were larger and richer in nutrients than the younger nymphs, so the female parasitoids needed more young nymphs to obtain nutrients for oogenesis. Additionally, the aphid’s defense reactions may lead to the preference of aphelinids for younger hosts [60,61,62]. Furthermore, the average number of *A. gossypii* infesting cucumbers (*Cucumis sativus*) and killed by *A. asychis* by non-reproductive host killing was 73.9 [63]. As shown in our study, an increase in the rearing temperature significantly decreased the *C*_0_, and the regression equation for *C*_0_ and temperature showed a similar decreasing tendency from 20 to 30 °C. However, the *g_x_* showed an irregular variation in the adult stage at all four constant temperatures, and it was higher at mid-term than prophase. This phenomenon indicated that the emergence rate for progenies in the next generation was influenced by female adult age, which was also found in our previous study [13]. In addition, the *N*_0_ of aphids killed by *A. asychis* decreased significantly with increasing temperature in this study, which suggested that *N*_0_ might be influenced by temperature.

In this study, the *r* of *A. asychis* in the 24 and 28 °C treatments were significantly greater than those in the 20 and 32 °C treatments, which suggested that increasing the temperature benefited the *r*, but a further increase in temperature negatively affected the population parameter. The *r* of *A. asychis* feeding on *A. gossypii* nymphs was 0.255 at 25 °C [55], which was much greater than that at 20 to 32 °C in our study. The differences between the two studies might be influenced by the host species and temperature.

It is well known that the ability of a natural enemy to kill a pest in a lifespan partially represents its control efficiency. For the age-stage distribution of a stable population, the *θ* was used to compare the control potential of the natural enemy [43,64]. Our research results showed that the *θ* of *A. asychis* gradually decreased significantly with increasing temperature. Therefore, the killing potential of *A. asychis* for *M. persicae* on chili peppers was affected by temperature, but the most suitable temperature was around 24 °C. 

*Aphelinus asychis* has a wide distribution in Asia, Europe, and North and South America [65], and has been used in Russia, China, South Korea, Japan, and America [13,17,22,59,66], but the climates in these regions and countries vary. Kalinkat et al. suggested that climate change may influence the functional responses of parasitoid–host pairs via temperature [67]. Temperature is the primary abiotic factor of climate change, which may affect insect development, reproduction, parasitizing behavior, distribution range, and biological clock [68,69,70,71]. The capacities of insects to adapt to new environmental conditions might be conferred by either plasticity or genetic evolution [37]. The difference in the population fitness of *A. asychis* at the four constant temperatures might be related to a similar mechanism. However, the exact mechanism of adaptation to these circumstances in *A. asychis* remains unclear and should be studied in the future. In addition, the control efficiency of *A. asychis* for *M. persicae* under greenhouse and field conditions, in which the temperature could be varied greatly, need to be tested in future research.

## 5. Conclusions

*Aphelinus asychis* could develop from egg to adult and reproduce successfully within a temperature range of 20–32 °C. The intrinsic rate of increase (*r*) of *A. asychis* at 24 and 28 °C was greater than that at 20 and 32 °C, and the finite aphid killing rates (*θ*) at 24, 28, and 32 °C were better than that at 20 °C. The population projection showed that *A. asychis* increased much faster at 24 and 28 °C. The results of fitting data showed that the temperatures for the expected maximum intrinsic rate of increase (*r*_max_) and the maximum finite killing rate (*θ*_max_) were 25.7 and 27.4 °C, respectively, which suggested that the most suitable range of temperatures for *A. asychis* for controlling *M. persicae* in chili pepper fields might be between 25.7 and 27.4 °C. 

## Figures and Tables

**Table 1:** Population parameter definitions and formulas used in the computer programs including TWOSEX–MSChart, CONSUME–MSChart, and TIMING–MSChart.

Parameter	Definition	Formula
sxj	Age-stage-specific survival rate	$s_{xj}=\frac{n_{xj}}{s_{01}}$
lx	Age-specific survival rate	$l_{x}=\overset{\beta }{\underset{j=1}{\sum }}s_{xj}$
mx	Age-specific fecundity	$m_{x}=\frac{\sum_{j=1}^{\beta }s_{xj}f_{xj}}{\sum_{j=1}^{\beta }s_{xj}}$
r	Intrinsic rate of increase	$\lambda =e^{r}$
R0	Net reproductive rate	$R_{0}=\overset{\infty }{\underset{x=0}{\sum }}l_{x}m_{x}$
T	Mean generation time	$T=\frac{\ln\left(R_{0}\right)}{r}$
kx	Age-specific host feeding rate	$k_{x}=\frac{\sum_{j=1}^{\beta }s_{xj}c_{xj}}{\sum_{j=1}^{\beta }s_{xj}}$
qx	Age-specific net host feeding rate	$q_{x}=l_{x}k_{x}$
C0	Net host feeding rate	$C_{0}=\overset{\infty }{\underset{x=0}{\sum }}l_{x}k_{x}$
ψ	Stable host feeding rate	$\psi =\overset{\infty }{\underset{x=0}{\sum }}\overset{\beta }{\underset{j=1}{\sum }}a_{xj}c_{xj}$
ω	Finite host feeding rate	ω=λφ
gx	Age-specific non-effective parasitism rate	$g_{x}=\frac{\sum_{j=1}^{\beta }s_{xj}d_{xj}}{\sum_{j=1}^{\beta }s_{xj}}$
hx	Age-specific net non-effective parasitism rate	$h_{x}=l_{x}g_{x}$
N0	Net non-effective parasitism rate	$N_{0}=\overset{\infty }{\underset{x=0}{\sum }}l_{x}g_{x}$
γ	Stable non-effective parasitism rate	$\gamma =\overset{\infty }{\underset{x=0}{\sum }}\overset{\beta }{\underset{j=1}{\sum }}a_{xj}d_{xj}$
ε	Finite non-effective parasitism rate	ε=λγ
μx	Age-specific aphid killing rate	$\mu_{x}=\frac{\sum_{j=1}^{\beta }s_{xj}p_{xj}}{\sum_{j=1}^{\beta }s_{xj}}$
wx	Age-specific net aphid killing rate	$w_{x}=l_{x}u_{x}$
Z0	Net aphid killing rate	$Z_{0}=\overset{\infty }{\underset{x=0}{\sum }}l_{x}u_{x}=R_{0}+C_{0}+N_{0}$
ϑ	Stable aphid killing rate	$\vartheta =\overset{\infty }{\underset{x=0}{\sum }}\overset{\beta }{\underset{j=1}{\sum }}a_{xj}p_{xj}$
θ	Finite aphid killing rate	θ=λϑ
Qp	Transformation rate	$Q_{p}=\frac{Z_{0}}{R_{0}}=\frac{R_{0}+C_{0}+N_{0}}{R_{0}}$
p(t)	Population growth	$p\left(t\right)=\overset{m}{\underset{j=1}{\sum }}\left(\overset{\infty }{\underset{x=0}{\sum }}f_{xj}n_{xj,t}\right)$ t: the simulation time. m: number of life stages. nxj: number of individuals of age x and stage j.
v(t)	Killing potential	$v\left(t\right)=\overset{m}{\underset{j=1}{\sum }}\left(\overset{\infty }{\underset{x=0}{\sum }}p_{xj}n_{xj,t}\right)$

The age-stage-specific fecundity (*f_xj_*) was the number of parasitoid progeny at age *x* and stage *j*. The age-stage-specific host feeding rate (*c_xj_*) was the number of aphid nymphs killed by *A. asychis* female adults at age *x* and stage *j* for feeding. The age-stage-specific non-effective parasitism rate (*d_xj_*) was the number of aphid nymphs parasitized by *A. asychis* at age *x* and stage *j* but for which emergence failed. The age-stage-specific aphid killing rate (*p_xj_*) was the number of aphid nymphs fed on by *A. asychis* at age *x* and stage *j*, and the *p_xj_* value is the sum of *f_xj_*, *c_xj_*, and *d_xj_*.

**Table 2:** Development and fecundity of *A. asychis* parasitizing *M. persicae* on chili peppers at four constant temperatures.

Parameters	20 °C		24 °C		28 °C		32 °C	
	n	Mean ± SE	n	Mean ± SE	n	Mean ± SE	n	Mean ± SE
Emergence rate of the parent cohort (%)	43	86%	40	80%	40	80%	26	52%
Female proportion of the parent cohort (%)	43	74.4 ± 6.7% a	40	62.5 ± 7.7% ab	40	52.5 ± 7.9% bc	26	65.4 ± 9.3% ab
Female preadult duration (d)	32	20.1 ± 0.3 a	25	14.1 ± 0.2 b	21	9.5 ± 0.1 c	17	9.7 ± 0.2 c
Female longevity (d)	32	38.0 ± 2.1 a	25	26.6 ± 0.8 b	21	16.0 ± 0.7 c	17	14.0 ± 0.6 d
Male preadult duration (d)	11	20.4 ± 0.7 a	15	13.1 ± 0.2 b	19	9.6 ± 0.1 c	9	9.4 ± 0.2 c
Male longevity (d)	11	34.6 ± 0.9 a	15	23.4 ± 0.9 b	19	13.0 ± 0.3 c	9	11.7± 0.5 d
Reproduction period (d)	32	16.0 ± 2.0 a	25	11.5±0.8 b	21	5.9±0.6 c	17	4.2±0.6 d
Fecundity (progeny adults/female)	32	238.6 ± 31.0 a	25	150.3 ± 11.8 b	21	50.1 ± 5.9 c	17	11.7 ± 2.1 d

Different letters in the same row indicate significant differences (paired bootstrap test, *p <* 0.05).

**Table 3:** Population parameters, host feeding, non–effective parasitism and aphid killing of *A. asychis* parasitizing *M. persicae* on chili pepper at four constant temperatures.

Parameters	20 °C	24 °C	28 °C	32 °C
*r* (d^−1^)	0.1848 ± 0.0051 a	0.2360 ± 0.0082 b	0.2441± 0.0140 b	0.1676 ± 0.0186 a
*λ* (d^−1^)	1.2030 ± 0.0061 a	1.2662 ± 0.0103 b	1.2765 ± 0.0179 b	1.1825 ± 0.0219 a
*R*_0_ (progeny adults)	177.5 ± 27.6 a	94.0 ± 13.6 b	26.3 ± 5.0 c	7.6 ± 1.7 d
*T* (d)	28.0 ± 0.5 a	19.2 ± 0.2 b	13.4 ± 0.3 c	12.1 ± 0.2 d
*C*_0_ (aphids)	26.6 ± 4.0 a	18.2 ± 2.7 b	6.9 ± 1.4 c	3.0 ± 0.6 d
*N*_0_ (aphids)	18.7 ± 3.0 a	11.9 ± 1.7 a	5.5 ± 1.3 b	3.3 ± 0.8 b
*Z*_0_ (aphids)	222.8 ± 34.4 a	124.0 ± 17.4 b	38.6 ± 7.3 c	14.0 ± 2.9 d
*ϑ*	0.2610 ± 0.0145 a	0.3550 ± 0.0235 b	0.4241 ± 0.0412 b	0.3647 ± 0.0371 b
*θ*	0.3140 ± 0.0190 a	0.4495 ± 0.0333 b	0.5414 ± 0.0595 b	0.4312 ± 0.0511 b
*Q_p_*	1.2552 ± 0.0124 a	1.3201 ± 0.0166 b	1.4691 ± 0.0429 c	1.8333 ± 0.0680 d

Different letters in the same row indicate significant differences (paired bootstrap test, *p <* 0.05).
